# Experimental Study on the Incipient Movement of Muddy Clay under Different Salinity Conditions

**DOI:** 10.1155/2022/5245928

**Published:** 2022-09-05

**Authors:** Xiaolei Zhang, Xin Liu, Haoran Wu, Shuyu Liu, Yu Zhu, Zhengzheng Bi, Zhiheng Xu

**Affiliations:** ^1^School of Water Conservancy, North China University of Water Resources and Electric Power, Zhengzhou, Henan 450046, China; ^2^Lijin Bureau, Estuary Bureau, Yellow River Conservancy Commission, Dongying, Shandong 257000, China

## Abstract

In order to understand the incipient movement of muddy clay under different salinity conditions, three series of flume tests were performed on incipient movement of muddy clay, including tests on incipient movement of salt-free clay mud under salt water conditions (salt water-salt-free clay mud), incipient movement of salt clay mud under salt water conditions (salt water-salt clay mud), and incipient movement of salt clay mud under freshwater conditions (freshwater-salt clay mud), using a circulating flume, in which the salinity of the water body or cohesive sediment varies from 0 to 40%. Based on the particle image velocimetry system and digital image gray processing technology, the gray curves of water near the clay mud bed surface with the velocity were plotted to quantitatively differentiate the incipient velocity of the sediment for each test. The experimental results showed that the higher the salinity of the water body or cohesive sediments is, the more difficult it is to start moving. There is a logarithmic relationship between the incipient velocity of cohesive sediments and the salinity of the water or cohesive sediments. The incipient velocity increases sharply at a salinity of 0∼10% and slowly at a salinity of 10∼40%. At the same salinity, the incipient velocity of salt clay mud under freshwater conditions is the largest, followed by that of salt clay mud under salt water conditions, while that of salt-free clay mud under salt water conditions is the smallest. In addition, the flow turbulence characteristics were analyzed under the critical conditions of the onset of muddy clay. Ultimately, an empirical formula to calculate the critical incipient velocity of muddy clay is proposed by introducing the salinity. In this study, salinity is included as a reference variable, which expands the research scope of sediment initiation and provides a reference for the study of estuary dynamics.

## 1. Introduction

Under the dual effects of runoff discharge and ocean tidal current uptracking, both land and sea sediments infill estuaries, bays, and silty coasts. After long-distance sorting and transportation to the estuary and coast, the riverine sediments are very fine. The grain size of the offshore sediments lifted by the wind and waves and then brought into the estuary and coast with the tide is very small. When the particle size of the sediment is small to a certain extent, it belongs to the category of cohesive sediment. Most of the sediments in estuaries and silty coasts belong to this category. Affected by runoff discharge, tidal current, and seasonal climate, the cohesive sediments in these areas are often found in the water environment of changing salinity. Salt content in the water body can significantly increase the flocculation and agglomeration characteristics of cohesive sediments, which has an important impact on the incipient movement of the sediment, flow and sediment transport, fluvial process, and pollutant diffusion in the area [1, 2]. Therefore, the research on the incipient movement of cohesive sediment is the key to solving the engineering sediment problem, which has important practical implications [3–9].

The current research on the incipient movement of cohesive sediment conducted experiments by either making theoretical derivation from the perspective of stress, considering the single variable that affects the physical index of the incipient movement of cohesive sediment, or making field measurements according to the actual engineering situation. These are briefly reviewed in this section.

Fortier et al. [10] studied the effect of mean particle size on the incipient movement of a mixture of silty loam and clay. Kamphuis et al. [11] experimentally studied the effect of variations of clay content and consolidation pressure on the critical shear stress of cohesive sediment. Kothyari et al. [8] also studied the effect of varying clay proportions, ranging from 10 to 50%, on the incipient movement characteristics of cohesive sediment mixtures. Aberle et al. [12] experimentally studied the effects of bed material properties, such as dry bulk density, water content, organic content, and sand content, on cohesive sediment erosion. Based on a series of laboratory investigations, Mehta et al. [13] showed that erosion mechanisms are directly associated with the bed structure and found that the critical shear stress increased with both the shear strength and the bed consolidation time, and the rate of erosion decreased with increasing consolidation time. Hanson et al. [14] reported that the erosion resistance of given cohesive sediment significantly increases with the increase in dry density of that sediment. To understand the mechanism of incipient movement of cohesive sediments, several studies focused on the influence of water content, bulk density, or the strength of the cohesive sediments [15–19]. Several researchers have argued that, besides the bulk density and water content of sediment, the shear strength also plays an important role in determining the critical condition of incipient movement of cohesive sediments [11, 20–29].

The parameterization of the threshold natural cohesive-adhesive sediment movement based on moment balance and dimensional considerations was developed [30]. Jepsen et al. [31], Roberts et al. [32], and Pang et al. [33] independently proposed the relationship between the shear stress of cohesive sediment and the sediment bulk density. The bulk density as a function of depth in the sediments has also been determined for relatively undisturbed sediments. Lyle et al. [34] have proposed an empirical relationship to calculate the critical shear stress, as a function of the void ratio and other sediment properties, like plasticity index, vane shear strength, and percent clay, by using sediment from Texas soils. Tan et al. [35] experimentally studied the effect of variations of consolidation time and particle size on the scour rate of cohesive sediment and proposed a relationship to calculate the scour rate of cohesive sediment that depends on dry unit weight and particle size. Zhang et al. [36] conducted experiments to determine the relationship between the critical condition of incipient movement and the rheological properties of the cohesive sediments.

Ganaoui et al. [37] identified two major classes of eroded particles: the first one corresponds to a “fluff layer” that is mainly representative of recent deposits of suspended particles characterized by a very low critical erosion shear stress, and the other one corresponds to consolidated cohesive matter with a significantly larger critical erosion shear stress. Many in situ sediment flumes have been developed for the study of vertical profiles of the critical erosion threshold in sediment. Vertical profiles of the critical erosion threshold (tcrit) in sediment have been measured at 11 stations along the axis of the Tamar Estuary and at a single station in a tributary of the Tamar at St. John's Ford [38].

The research on the incipient movement of cohesive sediment has become relatively mature, but most of the currently established incipient movement equations are only applicable to the incipient movement of cohesive sediment in freshwater. Moreover, the research on the incipient movement and scour of the consolidated cohesive sediment (the sediment density is more than 1,600 kg/m^3^) has been more comprehensive. However, research studies on the incipient movement of cohesive sediment under the influence of salinity are rare. In fact, whether the salinity of water affects its turbulent characteristics and the mechanism by which salinity variation affects the incipient movement of cohesive sediment remain unknown. It is also unknown whether the influence of salinity on the incipient velocity of sediment is due to the effect of the change of salinity on hydrodynamic conditions or completely depends on the effect of salinity on the physical and chemical properties of fine sediment itself. All these unknowns need to be studied.

The purpose of this study is to measure and investigate the effects of salinity variation of the water body or cohesive sediments on the critical condition of incipient movement of cohesive sediments. Toward this end, laboratory experiments were performed in a circulating flume to investigate the critical condition. During these experiments, through the use of the laser source and CMOS camera of the particle image velocimetry (PIV) system and digital image gray processing technology, the incipient movement of the sediment was measured, and the critical incipient velocity of sediment was determined, thus avoiding the subjectivity of visual observation. In addition, the main mechanisms by which sediment is moved in flowing water were analyzed from the perspective of the velocity of flow, shear, and normal stress resulting from the turbulent flow.

## 2. Experimental Program

### 2.1. Test Sand Samples

The experimental sediment samples with a median particle size of 8 *μ*m were obtained from the Yellow River Huayuankou through screening and sedimentation. According to the classification standard of China's Hydrologic Engineering, the sediment mixtures were composed of 6.9% sand, 58.6% sandy silt, and 34.5% clay with a particle size of less than 5 *μ*m by weight, belonging to cohesive sediment, which can meet the requirements of the cohesive sediment incipient test. In the process of flocculation and settlement to the bed surface, the cohesive fine sediments gradually consolidate; when the bulk density is less than 1,200 kg/m^3^, it is fluid mud; when the bulk density is more than 1,200 kg/m^3^ and less than 1,600 kg/m^3^, it is muddy clay; when the bulk density is more than 1,600 kg/m^3^, it is consolidated clay; when the sediment bulk density reaches 1,300 kg/m^3^, the sediment belongs to muddy clay. In addition, the variation range of salinity of the sea area is generally less than 40%. Therefore, the experimental sediment samples with a salinity of 0, 5, 10, 20, 30, and 40% were prepared by stirring at the same speed of 520 r/min, and the bulk density of all was 1,300 kg/m^3^ (about 2 hours from the laying of the mud bed to the completion of the sediment incipient movement test, the sediment has been compacted to 1,350 kg/m^3^).

### 2.2. Experimental Setup

**Figure 1:**
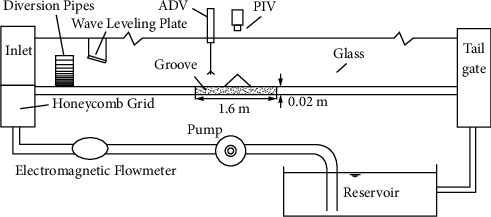
Sketch layout of experiment flume.

The experiments were conducted in a circulating flume measuring 16 m in length, 0.75 m in width, 0.5 m in depth, and a gradient of 5 in 1,000, with a rectangular cross section (Figure 1). In order to avoid the disturbance of the water flow caused by the inlet and the tailgate section of the flume and ensure constant and uniform turbulent development of the experimental measurement section, a groove measuring 1.6 m in length, 0.25 m in width, and 0.015 m in height was set at the middle section of the flume to lay sediment samples. The sediment samples were filled in the test section up to the level of the general flume bed. The surface of the mud bed and the joint between the mud bed and groove were smoothed with a scraper so as to avoid the terrain affecting the sediment movement. The bottom and side wall of the tank were made of glass, and the wall is thus smooth and flat, which is convenient for experimental observation. The honeycomb grid, overflow plate, diversion pipes, and wave leveling plate were successively set at the inlet of the tank to level off the flow and wave. The circulating water enters the tank in the form of overflow so as to make the water flow straight. Then, through the diversion pipes and wave leveling plate, the rhombic ripple on the water surface can be reduced to make the water surface stable, which enables the test to be performed under the condition of constant and uniform flow and produces representative research results. The water supply into the flume was increased next in small increments by regulating the pump power with the help of a frequency converter and by operating the tailgate until the condition of incipient movement was developed. The discharge flowing into the channel was measured with an electromagnetic flowmeter. The salinity of the test water and sand samples was measured with an electronic salinometer (WS-31), whose principle is that the conductivity of the sample increases with the increase of the salinity. The gray image of the water near the mud bed surface was collected by the PIV system, and the vertical distribution of threshold velocities was recorded by an Acoustic-Doppler Velocimeter (ADV).

### 2.3. Test Method

In order to understand the incipient movement of muddy clay under different salinity conditions and to determine whether the influence of salinity on the incipient velocity of sediment is due to the effect of the change of salinity on hydrodynamic conditions or completely depends on the influence of salinity on the physical and chemical properties of the cohesive sediment itself, the experimental water bodies with salinity proportion of 0, 5, 10, 20, 30, and 40% were prepared in turn, and the water depth of all tests was adjusted to 15.5 cm, the experiments were performed by controlling only the single variable of salinity. Three series of tests of incipient movement of muddy clay were performed, including tests of incipient movement of salt-free clay mud under salt water conditions (salt water-salt-free clay mud), of salt clay mud under salt water conditions (salt water-salt clay mud), and of salt clay mud under freshwater conditions (freshwater-salt clay mud), in which the salinity of the water body or cohesive sediment varied from 0 to 40% using a circulating flume.

**Figure 2:**
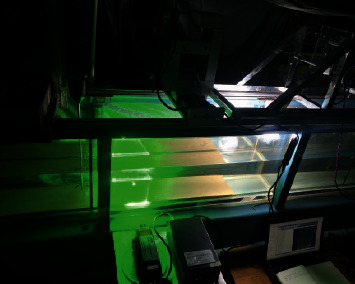
Gray image acquisition of sediment incipient movement.

In order to determine the critical initial condition of sediment, the gray level of water near the mud bed surface and the flow velocity were simultaneously measured during the onset. The laser and camera of the PIV system were installed in the middle of the mud bed. In order to collect gray images of the water near the mud bed surface during the onset, the laser system was placed horizontally, and the continuous light sheet source with a thickness of 1 mm formed by the laser system was adjusted as close to the surface of the mud bed as possible, which illuminated a very thin surface near the mud bed surface, and the camera lens was arranged perpendicular to the light sheet source (Figure 2). Then, based on the digital image gray processing technique, the gray level curve of the water near the mud bed surface with the velocity was plotted, and through the inflection point of the relation curve between the gray level of the water body and the velocity, the quantitative discrimination of the incipient velocity of sediment was achieved.

## 3. Results and Analysis

### 3.1. The Phenomenon and Discrimination of Muddy Clay Onset

In this study, the general onset of muddy clay was taken as the critical initial condition of sediment. First, the mud bed surface was noticed to appear through the formation of potholes over the bed surface in multiple locations, then those potholes were torn and elongated along the longitudinal direction, and the sediments from the locations of the potholes were carried away by the current in a smoky form. In the experiments, the threshold velocity (represented by the average velocity of the cross section) was determined by visual observation combined with the relationship between the gray level of water near the mud bed surface and the flow velocity during the onset.

**Figure 3:**
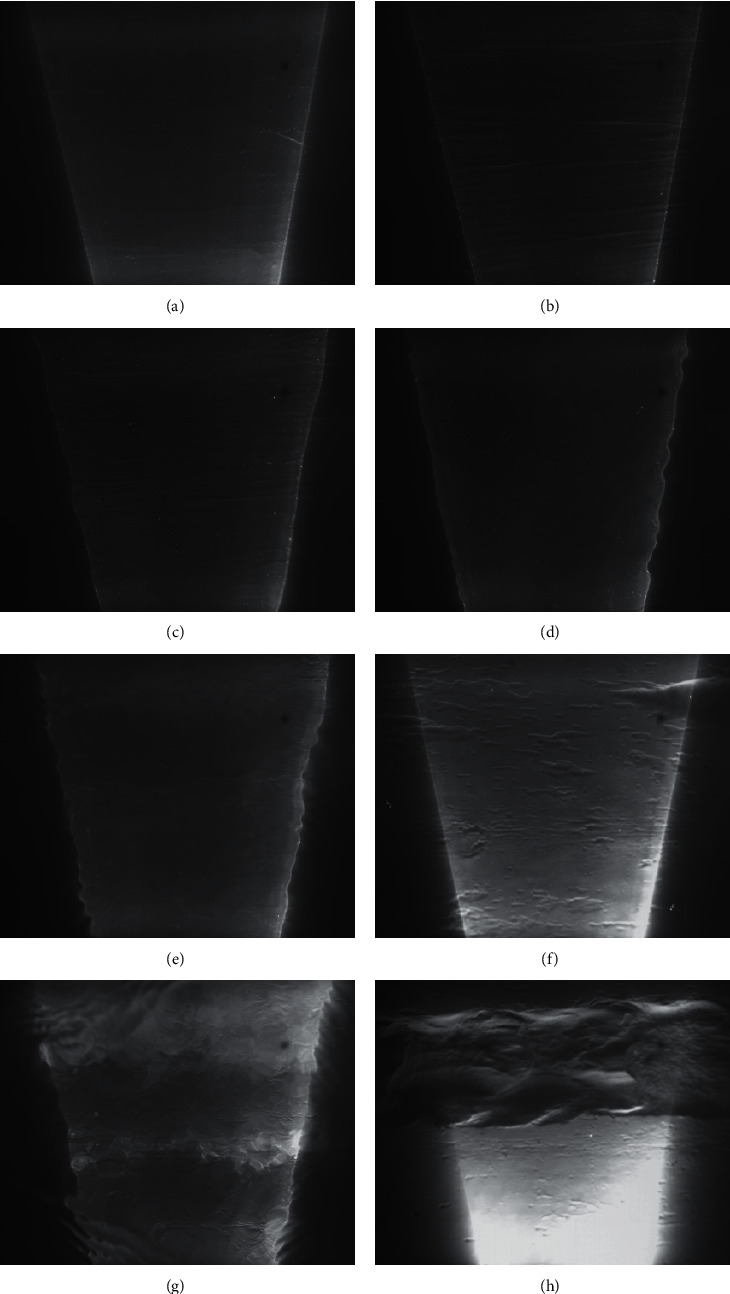
The phenomenon of muddy clay onset (10% salt water-0% salt clay mud). (a) The granular flocs. (b) Wispy stripes. (c) Dense stripes. (d) Sediment transport almost stops. (e) Sediment transport in the form of smoky. (f) Bed surface after sediment incipient movement. (g) Local scour. (h) Bed surface after scouring.

Taking 10% salt water-0% salt clay mud incipient movement test (the muddy clay incipient movement flume tests of sand samples with 0% salt under the condition of 10% salt water) as an example, the phenomenon of the onset of muddy clay is shown in Figure 3. When the flow velocity is low, the granular flocs attached to the mud bed surface move slowly with the flow, as shown in Figure 3(a). With the increase of the flow velocity, the granular flocs create wispy stripes, as shown in Figure 3(b). With another increase in the velocity, the length of the stripes increases, the distance between the stripes also increases, and the velocity of sediment transport increases, as shown in Figure 3(c). When the velocity increases to a certain value, sediment transport almost stops, and the bed surface becomes smooth and clean, as shown in Figure 3(d). When the velocity increases to a certain value again, small potholes appear in the weak position of the bed surface, and sometimes a small number of thin flakes of sediment are carried away by the current. When the flow velocity increases further, the detachment begins by removing thin flakes from the bed surface, and continuously a number of thin flakes of sediment from the locations of potholes are carried away by the current. When the velocity increases slightly, those potholes are torn and spread both longitudinally and laterally, but more in the longitudinal direction, and the sediment is transported in a smoky form in many places of the bed, which indicates that the sediments are generally initiated into movement as shown in Figures 3(e) and 3(f). In particular, in the saline water environment, the cohesive sediment particles are more easily flocculated, sediment floc structure is more stable, the bed surface shear resistance is enhanced, the velocity required for sediment incipient movement is increased, and the weak position of the bed surface is prone to scouring, as shown in Figures 3(g) and 3(h). With the increase of the velocity, either the bed surface does not move or the incipient movement causes the formation of local scouring.

**Figure 4:**
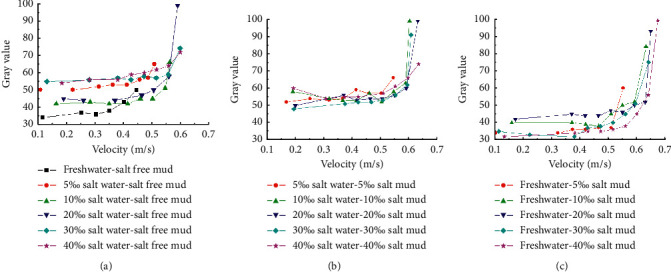
The gray level variation of water with flow velocity during the muddy clay onset. (a) Salt water-salt-free clay mud. (b) Salt water-salt clay mud. (c) Freshwater-salt clay mud.

The gray level variation of water with the flow velocity during the onset of muddy clay is shown in Figure 4. The gray level of water close to the bed surface is exponentially related to the velocity; the gray level first increases slowly and then increases sharply. With a different gray level of water before each experiment, when the salinity of the water body is high, it is impossible to use the same gray level to distinguish the incipient velocity of sediment in each experiment; thus, the velocity corresponding to the abrupt increase of the gray level is regarded as the incipient velocity. Before the sediment starts moving, the gray level of the water occasionally fluctuates through the increase of the flow velocity, which corresponds to the sediment initial movement. When the floating mud on the bed surface is carried away by the current, the gray level of the water decreases. With the increase of the velocity, the thin flakes of the sediment are lifted and suspended by the current. The water becomes muddy, and the gray level increases. Under the condition of saline water or sediment, the gray level of the water does not change significantly before the flow velocity reaches the critical condition of sediment incipient movement. The gray level of the water increases sharply when sediments start moving, especially in the sediment incipient movement flume tests of salt clay mud under freshwater conditions (freshwater-salt clay mud); the gray level of the water under the critical condition of the muddy clay is twice of that before the sediment initial movement. This indicates that, under the condition of saline water or sediment, the low flow rate is more conducive to the flocculation and agglomeration of cohesive sediments, the shear resistance of the sediment is enhanced, and it is not easy to start moving, when the velocity increases to the incipient velocity, the floc structure of cohesive sediments is destroyed, and then either the sediment starts moving or starting causes the formation of local scouring.

### 3.2. Influence of Salinity on Incipient Movement

**Figure 5:**
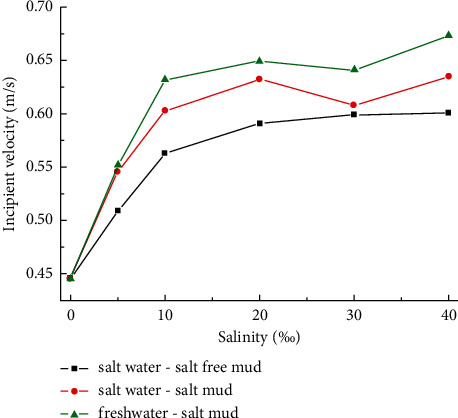
The variation of incipient velocity with salinity.

The incipient velocity of sediments under different salinity conditions was determined. The sediment samples from the Yellow River Huayuankou with 34.5% clay showed the characteristics of cohesive sediments. As shown in Figure 5, the higher the salinity of the water body or cohesive sediment is, the more difficult it is to start. There is a logarithmic relationship between the incipient velocity of cohesive sediments and the salinity of water or sediment. The incipient velocity increases sharply at the salinity of 0∼10% and slowly at the salinity of 10∼40%. For the same salinity, the incipient velocity of salt clay mud under freshwater condition is the largest, followed by that of salt clay mud under salt water condition, and that of salt-free clay mud under salt water condition is the smallest.

Salinity can enhance the effect of the driving force of water flow on the incipient movement of sediment and is also favorable to flocculation and agglomeration of cohesive sediment. With the increase of salinity, the cohesive force between particles increases, the floc structure is more stable, and the shear resistance of the mud bed is increased; thus, the velocity required for the incipient movement of sediment increases. However, after about 2 hours of the experimental stage, the sediment cohesive force failed to reach the maximum.

For salt water-salt-free clay mud, the salinity of the test sand sample is due to the gradual infiltration of salt water, which was initially admitted into the flume at a small discharge during the test; as a result, the salinity of the sand sample is slightly lower than that of the salt water. However, for salt water-salt clay mud, the salinity of the test mud, which is prepared by mixing and stirring with salt, is equal to that of the salt water; thus, at the same salinity, the incipient velocity of salt water-salt-free clay mud is lower than that of salt water-salt clay mud. In addition, according to a study by Wang et al. [39] on the rheological properties of cohesive sediment, the stirring speed of 520 r/min, which is used for mixing and stirring the experimental sand samples with saline, will destroy the stable structure of sand samples and reduce the viscosity of cohesive sediment in the present study. However, for the sediment from the Yellow River Huayuankou, the clay content is as high as 34.5%, and the effect of the enhancement of salinity on sediment viscosity is greater than the effect of the stirring speed-induced destruction of the stable sand structure on sediment viscosity. In other words, in the game between salinity and stirring speed, salinity wins, which is also the reason for the incipient velocity of salt water-salt clay mud being higher than that of salt water-salt-free clay mud. The comparison of the experiments with freshwater-salt clay mud and salt water-salt clay mud revealed that although the mud samples used in the test are the same, the buoyancy of the salt water is greater than that of the freshwater, and the lifting force acting on the cohesive sediment is stronger. Therefore, under the same salinity condition, the incipient velocity of salt water-salt clay mud is less than that of freshwater-salt clay mud. Moreover, according to the principle of “like dissolves like,” salt water penetrates more easily into the salt water molecules between salt clay mud particles than freshwater, so it is easier to drive the cohesive sediment flocs to start moving.

### 3.3. Turbulence Characteristics of Flow under Critical Condition for the Onset of Muddy Clay

#### 3.3.1. Probability Distribution of Turbulence Velocity

**Figure 6:**
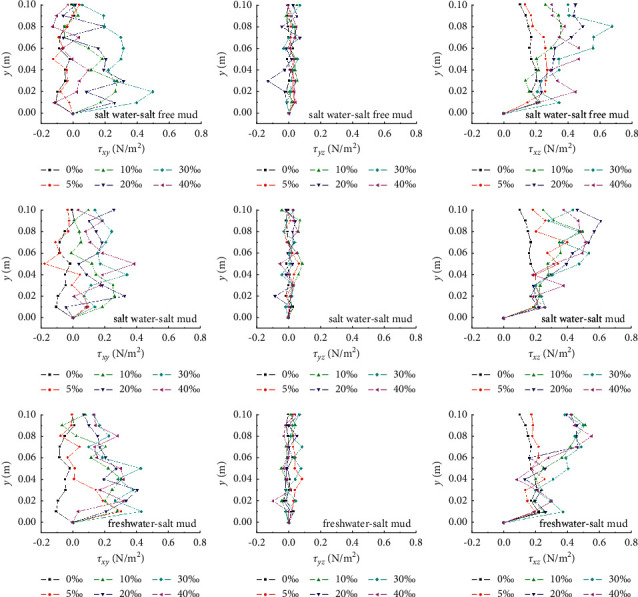
Turbulent stress of flow during the muddy clay onset.

The statistical analysis of the longitudinal, transverse, and vertical turbulent velocities (*u*_*x*_′*u*_*y*_′*u*_*z*_′) obtained from each measuring point on the vertical line showed that the turbulence velocity variables were arranged in order of magnitude and grouped according to a certain velocity interval. The number of times a variable appears in each group divided by the total number of times is called frequency. The frequency of the velocity in each velocity interval is calculated, and the empirical frequency curve, which is plotted in Figure 6, reveals that the probability density function of turbulent velocity approximately obeys normal distribution, and the probability density function is expressed by equation (1).

(1) $$\begin{array}{c} f\left(u_{i}^{\prime }\right)=\frac{1}{\sqrt{2\pi }\sigma_{u_{i}^{\prime }}}e^{-\left({\left(u_{i}^{\prime }-\bar{u_{i}^{\prime }}\right)}^{2}/2\sigma_{u_{i}^{\prime }}^{2}\right)}, \end{array}$$

where *f*(*u*_*i*_′) is the probability density function of turbulence velocity *u*_*i*_′ (*i* = *x*,*y*,*z*), $\bar{u_{i}^{\prime }}$ is the time mean value of turbulence velocity, and *σ*_*u*_*i*_′_ is the mean square error of turbulent velocity.

**Figure 7:**
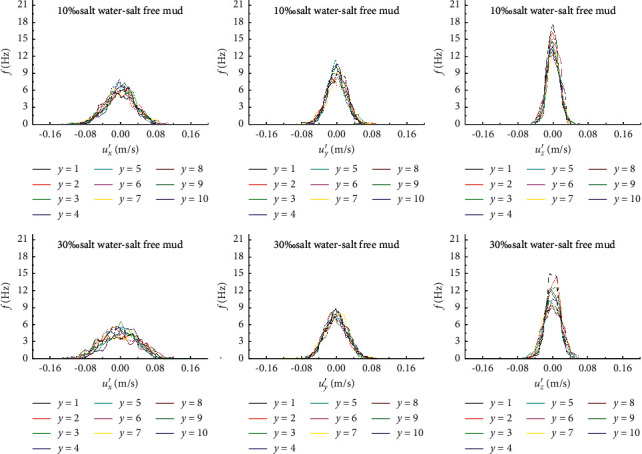
Probability distribution of turbulence velocity under critical condition of the muddy clay onset.

The incipient velocity of 10% salt water-0% salt clay mud and 30% salt water-0% salt clay mud is taken as an example in Figure 7, in which the ordinate *y* represents the relative height of the measuring point from the bed surface. The turbulence velocity in the three directions of each measuring point on the vertical line basically follows the normal distribution with zero mathematical expectation. The comparison of the frequency distribution curves of turbulence velocity in three directions reveals that the variation range of turbulence velocity is the largest in the longitudinal direction, followed by that in the transverse direction, and the smallest is in the vertical direction. The frequency distribution curve of turbulence velocity is “short and wide” in the longitudinal direction and “narrow and long” in the transverse direction. The vertical turbulence velocity fluctuates in a small range near zero, and the kurtosis is the largest. Along the vertical direction, the variation amplitude and kurtosis of the transverse and longitudinal turbulence velocity at each measuring point are staggered, and the frequency distribution curve is slightly “negatively skewed” or slightly “positively skewed” without an obvious change rule. However, the kurtosis of the vertical turbulence velocity decreases with the increase of the distance from the bed surface within the range of 4 cm and changes little with the increase of the distance from the bed outside the range of 4 cm. With the increase of the water or sediment salinity, the incipient velocity increases, the variation range of turbulence velocity in all directions increases, and the kurtosis value decreases, especially when the transverse turbulent velocity frequency distribution curve is “shorter and wider.”

#### 3.3.2. Turbulence Intensity

The mean square root of turbulence velocity *u*_*i*_′ (*i* = *x*, *y*, *z*) is used to express the turbulence intensity *σ*_*u*_*i*__, which is shown in equation (2). It reflects the intensity of turbulence at different positions of flow.

(2) $$\begin{array}{c} \sigma_{u_{i}}=\sqrt{\bar{u_{i}^{\prime 2}}}. \end{array}$$

**Figure 8:**
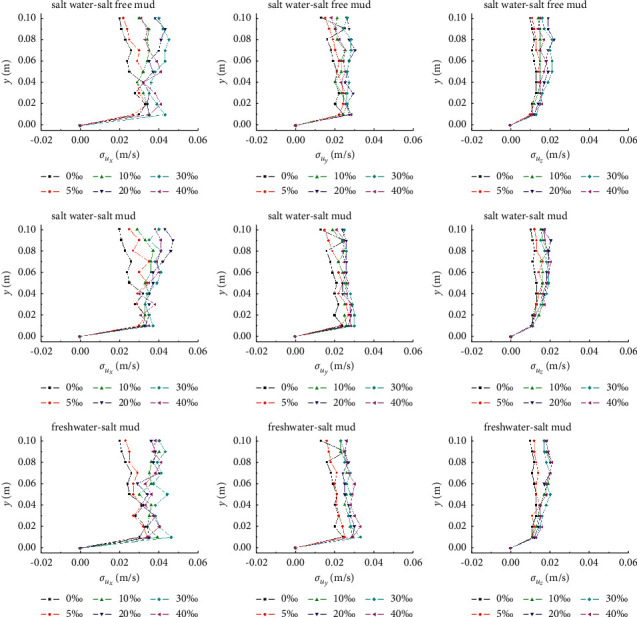
Turbulence intensity of flow under critical condition of the muddy clay onset.

The turbulence intensity curves of the flow under the critical condition of the onset of the muddy clay displayed in Figure 8 indicate that, with the increase of salinity, the incipient velocity increases, and the turbulence intensity of the flow in all directions basically increases. In addition, the vertical distribution of turbulence intensity shows that the longitudinal (along the flow direction) turbulence intensity is the largest, followed by the transverse, and the vertical (along the water depth direction) turbulence intensity is the smallest. Moreover, longitudinal turbulence intensity increases greatly with the increase of salinity and velocity above 0.04 m from the bed surface, and the transverse turbulence intensity decreases slightly from bottom to top vertically. The vertical distribution of vertical turbulence intensity shows an exponential trend of gradually increasing. The results also show that the vertical distribution trend of turbulence intensity of the onset of the muddy clay under the influence of salinity is divided by salinity of 10%. In particular, for the test results of the series of freshwater-salt clay mud, the vertical distribution of turbulence intensity of the onset of the muddy clay is clearly similar when the salinity of cohesive sediment is 0 and 5% and similarly when the salinity ranges from 10 to 40%, which is consistent with the variation of the incipient velocity of cohesive sediment with salinity.

#### 3.3.3. Turbulent Shear Stress

Turbulent shear stress is used to characterize the effect of turbulence on average movement. It is additional shear stress caused by the momentum exchange of turbulent microclusters between adjacent laminar flows. Its expression (equation (3)) is as follows:

(3) $$\begin{array}{c} \tau_{ij}=-\rho \bar{\overset{\prime }{\underset{i}{u}}\overset{\prime }{\underset{j}{u}},} \end{array}$$

where *τ*_*ij*_ is the turbulent shear stress and *ρ* is the density of water. When *i* = *j*, *τ*_*ij*_ is the normal turbulent stress, and when *i* ≠ *j*, *τ*_*ij*_ is the turbulent shear stress. The calculation formulas of turbulence intensity and normal turbulent stress show that the distribution law of normal turbulent stress is the same as that of turbulence intensity. Therefore, only the distribution law of turbulent shear stress is discussed in the following.

As shown in Figure 6, in absolute terms, *τ*_*xz*_ is the largest, followed by *τ*_*xy*_, and *τ*_*yz*_ is the smallest. The results show that *τ*_*xy*_ oscillates sharply between positive and negative directions and generally increases with salinity and velocity; *τ*_*yz*_ changes little along the vertical direction, and its absolute value is close to zero. The vertical distribution rule of *τ*_*xz*_ is basically consistent with those of longitudinal turbulence intensity, which is related to the smaller variation of vertical turbulence along the water depth.

#### 3.3.4. Turbulent Kinetic Energy

Turbulent kinetic energy (*E*) is a characteristic value reflecting the energy carried by turbulent flow, which is expressed by equation (4).

(4) $$\begin{array}{c} E=\rho *\frac{\left(\bar{u_{x}^{\prime 2}}+\bar{u_{y}^{\prime 2}}+\bar{u_{z}^{\prime 2}}\right)}{2}. \end{array}$$

**Figure 9:**
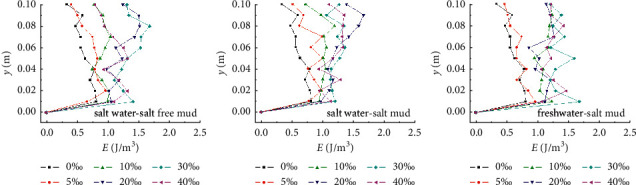
Turbulent kinetic energy of flow under critical condition of the muddy clay onset.

As shown in Figure 9, with the increase of salinity, the incipient velocity of sediment increases and the turbulent kinetic energy of flow basically increases. The vertical distribution rule of turbulent kinetic energy is well consistent with those of longitudinal turbulence intensity. It is clear that the longitudinal turbulence is the largest, and the transverse and vertical turbulences are small and distributed uniformly along the vertical direction.

## 4. Discussion

### 4.1. Comparisons

**Figure 10:**
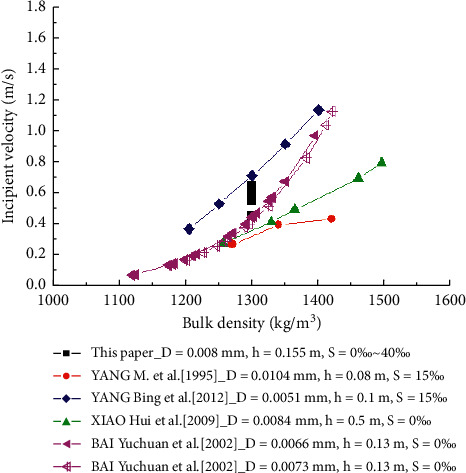
Sediment-moving incipient velocity from lab.

The comparison of the critical incipient velocity values of salt clay mud under saline conditions measured in this study and plotted in Figure 10 with those of other muddy clay incipient tests [40–43] reveals a tendency of the measured values of incipient velocity that is consistent with the reported results [42, 43]. The values measured in fresh water by Bai et al. [43] increased exponentially with the bulk density of sediment samples. The velocity values measured in 15% salt water environment by Yang et al. [42] were greater than the velocity values measured by Bai et al. [43] and increased linearly with the bulk density of sediment samples. When the bulk density reached 1,400 kg/m^3^, the measured values are close to each other. The tendency of these two curves is the same as the tendency of viscosity of muddy clay with sediment bulk density in saline water and fresh water [44]. As a result of this tendency, the viscosity of cohesive sediment increases with the increase of sediment bulk density, and the viscosity under salt water is greater than that under fresh water, and when the sediment bulk density is 1,400 kg/m^3^, the viscosity of cohesive sediment under salt water and fresh water is the same. The values of incipient velocity in the present experiments are nonunique for a certain bulk density. The measured values of incipient velocity are in a band that encompasses the above-mentioned two curves, as the variation of salinity. In the absence of salt, the measured values in our study are equal to those of the Bai et al. [43] measurements, and the maximum velocity corresponding to maximum salinity of 40% is slightly less than the measured value by Yang et al. [42]. This is due to the finer particle size of the experimental sediment sample studied by Yang et al. [42] compared to that in our study. Additionally, the increase rate of the velocity with salinity is lower when the salinity is greater than 10%. However, the measured results in our study are not consistent with those of Yang et al. [40] and Xiao et al. [41]. The velocity values measured by Yang et al. [40] are very low and increase logarithmically with the sediment bulk density due to the 5% content of gravel in the cohesive sediment mixtures studied by Yang et al. [40]. The median particle size of the sand sample tested by Xiao et al. [41] is the same as that in our study, and the test water depth is as high as 0.5 m, but their measured velocity values are clearly lower than those in our study. Additionally, their values increase linearly with the sediment bulk density, which is inconsistent with the tendency of the velocity of cohesive sediment with bulk density. In fact, their test sand sample belonged to the category of silty sand.

### 4.2. Empirical Formula for Critical Incipient Velocity of Muddy Clay

In order to make the test results more applicable, the formula of Yang et al. [40] for incipient velocity was improved. Based on the experimental data presented in this study and the published results [42, 43] in which the tendency is basically the same as that in this study, the parameters in the improved formula are obtained by linear fitting, and the incipient velocity formula of cohesive sediment with salinity variable is established.

Yang et al. [40] derived the cohesive shear stress based on the electrochemical theory of cohesive fine sediment flocculation and considered bulk density as a comprehensive index influenced by the characteristics of the muddy clay itself and the surrounding environmental factors, which can better reflect the change of the incipient velocity of muddy clay. The formula is as follows:

(5) $$\begin{array}{c} U_{c}=\frac{1}{\kappa }\text{ln}\left(11\frac{h}{k_{s}}\right){\left[\theta_{m}\frac{\gamma_{s}-\gamma }{\gamma }g\;D+C{\left(\frac{\gamma_{s}^{\prime }}{\gamma_{s^{*}}}\right)}^{n}\frac{1}{\gamma D^{q}}\right]}^{1/2}, \end{array}$$

where *U*_*c*_ is the incipient velocity; *κ* = 4 is the Karman constant; *h* is the water depth, *k*_*s*_ is the bed surface roughness height; *θ*_*m*_ is the Shields parameter under the critical condition of sediment incipient motion; *γ*_*s*_ is the solid bulk density of sediment; *γ* is the bulk density of clean water; *D* is the particle size of sediment; *C* is the coefficient considering the bonding force; *γ*_*s*_′ is the bulk density; *γ*_*s*^*∗*^_′ is the stable bulk density of sediment after compaction, taking 1,600 kg/m^3^; *n* is the index of the relative concentration related to the adhesion force; *q* is the coefficient related to the film water thickness on the particle surface.

Equation (5) is simplified by neglecting the first term of gravity. Also, since the incipient condition of cohesive sediment is different with the same median particle size but different particle composition, the clay content is more representative than the median particle size. Additionally, the particle size is replaced by the clay content *P*_*c*_, and then the salinity variable S is introduced; thus, the critical incipient velocity for muddy clay is considered to be mainly affected by the following:

(6) $$\begin{array}{c} U_{c}=f\left(h,\frac{\gamma_{s}^{\prime }}{\gamma_{s^{*}}^{\prime }},\gamma ,P_{c},S\right). \end{array}$$

The improved formula is as follows:

(7) $$\begin{array}{c} U_{c}=a\;\ln\left(11\frac{h}{k_{s}}\right){\left(\frac{\gamma_{s}^{\prime }}{\gamma_{s^{*}}^{\prime }}\right)}^{b}\gamma^{-\left(1/2\right)}P_{c}^{c}{\left(S*1000+0.1\right)}^{d}, \end{array}$$

where *a*, *b*, *c*, and *d* are undetermined coefficients.

After taking logarithm on both sides of equation (7) and linear fitting, the formula of incipient velocity of cohesive sediment under saline conditions obtained is shown in equation (8).

(8) $$\begin{array}{c} U_{c}=24.582\;\ln\left(11\frac{h}{k_{s}}\right){\left(\frac{\gamma_{s}^{\prime }}{\gamma_{s^{*}}^{\prime }}\right)}^{8.957}\gamma^{-\left(1/2\right)}P_{c}^{0.499}{\left(S*1000+0.1\right)}^{0.049}. \end{array}$$

**Figure 11:**
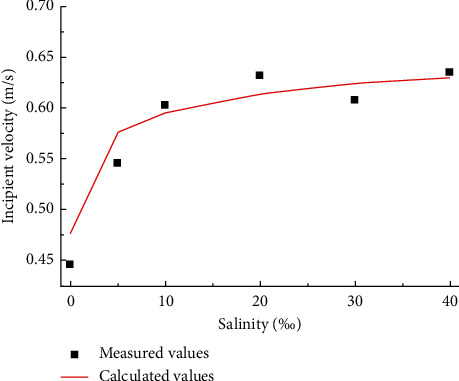
Comparison of calculated and measured critical incipient velocity of muddy clay.

**Figure 12:**
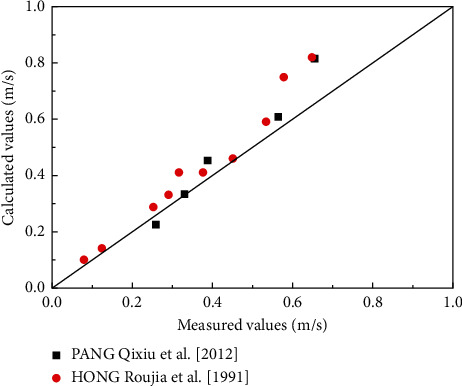
Verification of the empirical formula for incipient velocity of muddy clay.

As shown in Figure 11, the calculated values are in good agreement with the measured values in this study. The experimental data of Pang et al. [33] and Hong et al. [45] are used to verify the above-mentioned formula. The test sediment samples of Pang et al. [33] and Hong et al. [45] were collected from the clay mud of the Lianyungang main channel and Tianjin-Xingang, respectively, and had a clay content of 53 and 45%, respectively. The test water is salt water and fresh water, respectively. The comparison between the calculated values and the measured values, shown in Figure 12, reveals that when the incipient velocity is small, the calculated value of the formula is in good agreement with the measured value, and when the incipient velocity is large, the calculated value is slightly higher.

## 5. Conclusions

A study on the initial establishment of cohesive sediment under different saline conditions was performed in a circulating flume. Based on the PIV system and digital image gray processing technology, the quantitative discrimination of the incipient velocity of cohesive sediment is achieved. The flow turbulence characteristics are analyzed under the critical condition of the onset of the muddy clay. The results showed that the research results will contribute to enriching the theory of sediment initiation, revealing the mechanism of riverbed evolution, improving the river management system, and having important significance for shipping, flood prevention, ecological restoration, and other engineering problems. The results show the following:

1. When muddy clay is weakly incipient, the erosion rate of the mud bed is low, and the increase of the gray value of water near the bed surface is about 10. When muddy clay is generally incipient, the erosion rate of the mud bed increases significantly, and the gray value of the water body increases sharply, which can reach twice the value of that before the onset of the muddy clay.
2. The higher the salinity of the water body or cohesive sediment, the more difficult it is to start moving. There is a logarithmic relationship between the incipient velocity of cohesive sediment and the salinity of water or sediment. The incipient velocity increases sharply in the range of 0–10% and slowly in the range of 10–40%. For the same salinity, the incipient velocity of sand samples with salt under freshwater conditions is the largest, followed by that of sand samples with salt under saline conditions, and that of sand samples with a salinity of 0% under saline conditions is the smallest.
3. A relationship is proposed for the determination of the critical incipient velocity of muddy clay. The variables, namely, water depth, sediment bulk density, clay content, and salinity, are noted to be the main parameters controlling the incipient movement condition of muddy clay.
4. Under the action of uniform flow in a straight rectangular cross-section flume, the longitudinal turbulent fluctuation under the critical condition of the onset of the muddy clay is the largest, while the transverse and vertical turbulent fluctuation are smaller, and the distribution along the vertical direction is more uniform. The vertical distribution of *τ*_*xz*_ and turbulent kinetic energy are basically consistent with those of longitudinal turbulence intensity. The probability distribution of turbulent velocity is approximately normal. The vertical distribution of turbulence characteristics at the onset of the muddy clay under the influence of salinity is different with a salinity of 10% as the dividing line. Due to the limitations of the existing experimental conditions, this study can be conducted with multiple groups and in different areas in the future.

## Data Availability

Some or all data, models, or code that support the findings of this study are available from the corresponding author upon reasonable request. All data, models, and code generated or used during the study appear in the submitted article.
